# Balancing nutrition, ethics, and sustainability about dairy: UK consumers’ knowledge, attitudes, practice, and intended choices

**DOI:** 10.1017/jns.2026.10109

**Published:** 2026-07-07

**Authors:** Hattie Bracey, Michael Barnard, Solveig Mendowski, Jérémie Renaud, Stuart R. Gray, Emilie Combet

**Affiliations:** 1 Human Nutrition, School of Medicine, Dentistry and Nursing, College of Medical, Veterinary and Life Sciences, University of Glasgow, Glasgow, UK; 2 Aspens Services Ltd., Worcester, UK; 3 Valorex, La Messayais, France; 4 Bleu-Blanc-Coeur, Rennes, France; 5 School of Cardiovascular and Metabolic Health, College of Medical, Veterinary and Life Sciences, University of Glasgow, Glasgow, UK

**Keywords:** animal welfare, consumer attitudes, consumer behaviour, dairy products, food sustainability

## Abstract

Milk is nutritionally rich, but its production poses ethical and environmental concerns. Farming practices can influence milk’s nutritional quality and improve its sustainability, creating opportunities for innovation. This study explores consumer willingness to adopt improved products and the interrelationships of knowledge and attitudes towards the dairy industry in relation to purchasing and consumption. We conducted an online survey of UK adults from November 2021 to March 2022, covering knowledge and attitudes towards dairy, dairy consumption, likelihood to purchase improved milk, and socio-demographics. Participants were classified as low or high dairy consumers based on their intake: below or above 1.81 dairy portions per day. Of the 706 dairy consumers who completed the survey, 47% reported consuming ≥1.81 portions of dairy products daily. High dairy consumption predicted more positive attitudes towards dairy farmers than lower consumption. Conversely, low consumption was associated with greater concern for animal welfare and the environmental implications of dairy (*p* < 0.001). Price was the most important consideration when purchasing milk habitually; however, when presented with four different descriptions of milk, 41% of participants said they would definitely or very probably buy the product with improved trade, welfare, and sustainability standards, despite this product having the highest price per litre. Balancing the nutritional value of cows’ milk with the environmental consequences and ethical concerns of its production is a critical part of the debate to foster a food system that supports planetary and human health. Altering production methods and improving products can be part of the food system transformation.

## Graphical abstract

**Figure:**
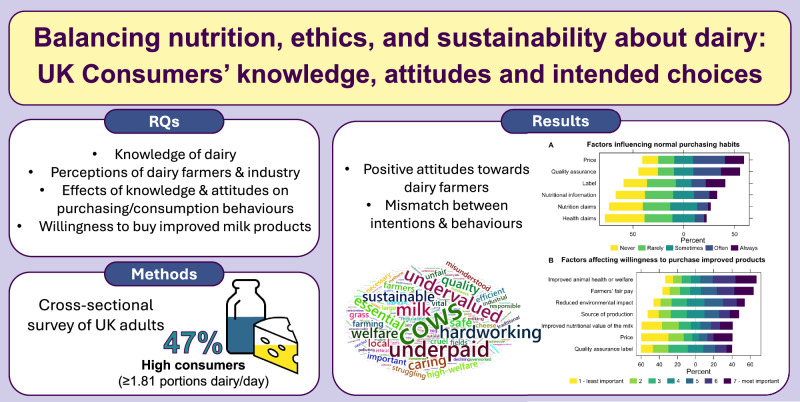

## Introduction

Cows’ milk makes a significant contribution to nutrient intake; it is a complete protein source and has a highly complex fat profile.^(1,2)^ Between 2018/19 and 2021/22, purchases of liquid milk fell by 11%, whilst plant-based alternatives (PBA) increased by 58%.^(3)^ Unlike PBA, supermarket own-brand products dominate cow’s milk purchases,^(4)^ indicating a substantial opportunity for innovation within this sector.

Milk’s complex matrix makes it an optimal vehicle for micronutrient enrichment, which can be altered during production or processing.^(5)^ The nutritional qualities of milk are intrinsically linked to cows’ diet; hence, dietary supplementation can be used to enrich milk with specific micronutrients or to alter the nutritional profile,^(6)^ such as shifting from saturated fatty acids towards polyunsaturated fatty acids.^(7)^ Alternatively, milk can be fortified with specific micronutrients (e.g. vitamin A, D, and iron) during processing. Some countries including Belgium and Sweden have mandated vitamin D fortification of certain dairy products, where fat-soluble vitamins are lost during processing.^(8,9)^ Whilst the UK does not have quantitative guidelines for dairy consumption, the Eatwell Guide suggests choosing dairy products with lower fat and sugar content, emphasising dairy as a source of protein and calcium. European guidelines suggest consuming 2–3 portions of dairy per day, equalling approximately 500 mL of milk.^(10)^ Among UK adults, dairy products account for 34% of calcium and 34% of iodine intake.^(11)^ Data from the 2014–2017 National Diet and Nutrition Survey (NDNS) showed that dairy consumers had a median urinary iodine concentration (UIC) of 132 µg/L, compared to 79 µg/L for those who exclusively consume PBA, indicating iodine insufficiency among non-dairy consumers, based on WHO criteria.^(12)^ The most recent NDNS data indicate that the median UIC has fallen to 109 µg/L for the population as a whole, and to 39 µg/L for people following vegan diets. This is of particular concern as specific population groups move away from cow’s milk due to concerns about health, animal welfare, and the environment.^(13,14)^ Changing consumer priorities, driven by health trends and environmental or ethical concerns, further provide an opportunity for market innovation.

Although the general public expresses support for British farmers,^(15)^ studies have highlighted the disconnect between consumers and producers owing to a lack of knowledge of agricultural practices^(16)^ and the environmental impact of our food choices.^(17,18)^ Dietary supplementation of dairy cows to improve feed efficiency and alter digestive processes can be an effective means of reducing enteric fermentation whilst altering the nutritional composition of milk, offering the potential to develop products with improved environmental and health parameters. Ascertaining the factors underpinning current consumer behaviours, driven by their attitudes and perceptions of dairy products and the industry is required to assess whether there is a market for such improved products. Whilst consumption data are well documented, more insight into the interaction between attitudes towards dairy and purchasing behaviours is needed. In this study, we assess the interrelationships between knowledge and attitudes towards the dairy industry and purchasing and consuming dairy products. Specifically, we aim to answer the following research questions: (i) What do UK dairy consumers know about dairy production and the nutritional qualities of milk? (ii) How do consumers perceive dairy farmers and the industry? (iii) How do knowledge and attitudes influence dairy purchasing behaviours? (iv) Is there a willingness among UK consumers to purchase milk with improved nutritional qualities and sustainability?

## Methods

### Study design

An online cross-sectional survey was conducted among UK adults between November 2021 and March 2022. Participants were recruited through social media advertisements and via the Prolific recruitment platform. Ethical approval for the study was granted by the University of Glasgow Ethics Committee (project no. 200210017). The proposed sample size was 800 UK adults, calculated from UK population data^(19)^ at a 95% confidence level (*α* = 0.05) and a 5% margin of error for each subgroup (low/high dairy consumers). We aimed to exceed this by 20% to account for incomplete or nonsensical responses, resulting in a final sample size of 960 respondents.

### Questionnaire

All data were self-reported and collected through the Qualtrics™ online survey platform. The survey comprised 61 questions (*n* = 150 total questions and sub-questions) across five sections assessing knowledge and attitudes about dairy products and the industry, attitudes towards purchasing milk products with different claims about nutrition, welfare, sustainability and trade standards, current dairy consumption habits, and socio-demographics (supplementary information (SI) 1). The survey design was informed by the knowledge–attitude–behaviour (KAB) model, which posits that combining an individual’s knowledge and attitudes towards a particular activity affects their behaviours. An extension of the KAB model is the theory of planned behaviour (TPB),^(20)^ which hypothesises that an individual’s intentions are influenced by their attitudes, subjective norms, and perceived behavioural control, and that these factors can predict whether they will engage with a particular behaviour. These models can be applied to ethical consumer behaviours by adding moral identity as a determinant of intention formation and moderation by the level of confidence in their attitudes and beliefs.^(21)^ The survey instrument was structured according to the KAB framework, with distinct sections assessing knowledge (Q1–14: factual understanding of dairy production, animal health, environmental impacts, and nutrition), attitudes (Q15–24: Likert-scale and word-association items on industry practices, animal welfare, and dietary perceptions), and behaviours/practices (Q27–39: self-reported consumption frequencies, purchase habits, and conjoint-based stated preferences). Elements of the TPB further informed specific items on behavioural intention (e.g. Q25–26: attitudes towards trying modified milks; Q38–39: purchase likelihoods via full-profile conjoint scenarios incorporating nutrition, environment, welfare, and price factors, implying perceived behavioural control through barriers like cost), though full TPB implementation (subjective norms and structural pathway modelling) was not pursued due to survey length constraints and participant burden.

### Knowledge

Multiple-choice questions (*n* = 12) with six options (including ‘*I don’t know*’) were used to assess knowledge about dairy farming, including animal husbandry (*n* = 6), milk processing (*n* = 2), environmental impacts (*n* = 2), physiological function of key nutrients (*n* = 4), and whether milk is a good source of specified micronutrients (*n* = 8) (available responses: ‘*not a source*’, ‘*is a source*’, ‘*I don’t know*’). Additionally, awareness and recognition of different quality assurance brands were assessed by asking participants to match logos to their brands (*n* = 3).

### Attitudes towards dairy

Attitudes were assessed using 5-point Likert scales (1 – ‘*strongly agree*’ to 5 – ‘*strongly disagree*’, or ‘*I don’t know*’) to statements (*n* = 23) about the dairy industry (*n* = 6), processors (*n* = 4), and farmers (*n* = 13) in the UK. Free-text responses allowed participants to provide three words associated with dairy foods and the dairy industry in a UK context and add any further comments.

### Dairy consumption

FFQ questions (*n* = 4), adapted from ^(22)^, assessed the consumption frequency of different types of milk (ruminant and PBA). Subsequent questions focused solely on cows’ milk, assessing the type of milk, frequency, and modes of milk consumption and other dairy products (including cheese, yoghurt, and dairy-based desserts). Likert scales (5-point, ‘*never*’ to ‘*always*’) provided insight into purchasing behaviours, including the type of retail outlet and considerations when buying dairy products.

### Likelihood to purchase improved milk

Participants were presented with a table describing four milk products labelled A–D (Table 1), with different attributes shown on their packaging. These attributes encompassed nutrition, animal welfare, sustainability, place of origin, quality assurance branding, fair price for farmers, and cost per litre, where prices were based on guidance from industry collaborators and the French market, where such products are available. Participants were asked how likely they would purchase each product (6-point Likert: ‘*definitely*’ to ‘*definitely not*’, and ‘*I don’t know*’) and which factors affected their decisions (7-point Likert: ‘*most important*’ to ‘*least important*’).

**Table 1.** Packaging descriptions for different milk products Table 1 long description.

	Product A	Product B	Product C	Product D
**Fortification**	Fortified with iodine, vitamin D, selenium, and omega-3 during processing	Cows fed a diet high in iodine, vitamin D, selenium, and omega-3 naturally enriching the milk	–	Cows fed a diet high in iodine, vitamin D, selenium, and omega-3 naturally enriching the milk
**Animal health and welfare**	–	Improved animal health and welfare	–	Improved animal health and welfare
**Sustainability/environment**	–	Reduced environmental impact	–	Reduced environmental impact
**Origin of product**	Produced locally/regionally	–	–	States production and processing locations
**Quality assurance branding**	Standard	Standard	Standard	Novel brand
**Fairtrade (price paid to farmers)**	–	–	–	Clear statement of farmers paid a fair price
**Cost per litre**	80p	90p	70p	£1

### Socio-demographics

In the final section, multiple-choice questions asked participants about their gender, age, ethnic background, partial postcode, education (attainment and subject area), household structure, employment status, household income, dietary pattern, health status, and household shopping and cooking habits.

### Analysis

Descriptive statistics were used to describe the population characteristics. For knowledge (Section 1, Q1–14), participants scored 1 point for correct answers and 0 for incorrect or ‘*I don’t know*’, except for identifying different quality assurance brands, for which participants scored 0.5 if two of three were correct, 1 point if all were correctly identified, or 0 if none or one were correctly identified. To compare the knowledge of different sections, cumulative scores were calculated for questions relating to i) the dairy industry, ii) nutrition, and iii) quality assurance branding; the maximum possible overall score was 23, and the minimum was 0. Attitudes (Section 2, Q15–24) towards dairy farmers were assessed using statements (*n* = 13) with a 5-point Likert-scale response (scored 1 to 5 points) and nine statements assessing attitudes towards the wider industry. Where respondents indicated a positive attitude (e.g. strong agreement with ‘*Dairy farmers in the UK care for their cows*’), responses were scored 5, whereas strong disagreement received 1. Where necessary, responses were reverse coded to align with the scoring. ‘*I don’t know*’ responses were removed from the analysis because they accounted for only a small proportion (<8%). Questions were categorised into four domains: environment/sustainability, animal welfare, nutrition, and UK food security (Table 2), from which cumulative scores (as percentages) were calculated. The validity of each composite score was assessed using Cronbach’s *α*; values ranged from 0.775 to 0.943, indicating acceptable validity. In the environment domain, higher scores indicated a perception that dairy production has a positive impact on the environment (e.g. increases biodiversity) and is not a major source of emissions. In the animal welfare domain, higher scores indicated favourable attitudes towards on-farm practices, for example, dairy farmers care for their cows, whilst lower scores indicated greater scepticism. Higher nutrition scores suggested that participants felt milk is a nutritious product and a key food for ensuring a healthy, balanced diet. In the food security domain, higher scores indicated an attitude that dairy production is important in ensuring UK food security. Sentiment analysis of free text responses was carried out using the ‘AFINN’ lexicon,^(23)^ numerically scoring individual words from −5 to + 5 based on their negative/positive connotations. Summing all the individual scores provides a final AFINN score; a higher score indicates greater positivity.

**Table 2.** Variables contributing to attitude scoring Table 2 long description.

UK food security
The dairy industry in the UK… …is an important factor in ensuring the UK produces its own food (Q15_5) …produces safer and higher quality food than in the EU (Q15_6) Dairy farmers in the UK… …contribute to the local and regional food landscape (Q19_1) …help ensure UK food security (Q19_2)
Animal welfare
Dairy farmers in the UK… …care for their animals/cows (Q17_1) …effectively manage herd health issues (Q17_2) …ensure that herd reproduction practices prioritise the welfare of both the calf and the cow (Q17_3) …responsibly manage the life outcomes of calves (Q17_4) …ensure their cows are living healthy and happy lives (Q17_5) …ensure their cows have a high-quality diet (Q18_1) …ensure their cows have plenty of access to outdoor space (Q18_2)
Nutritious product
Dairy processors in the UK… …want to create nutritious products for their customers (Q16_3) …support farmers to produce nutritious dairy products (Q16_4) Dairy farmers in the UK… …ensure the milk they produce is safe (Q18_3) …ensure the milk they produce is nutritious (Q18_4) …produce a key food to ensure people have a healthy, balanced diet (Q19_4)
Sustainability
The dairy industry in the UK… …is a major source of carbon dioxide and methane emissions (Q15_1, reverse coded) …is a major source of nitrogen emissions (Q15_2, reverse coded) …is doing significant damage to the land (Q15_3, reverse coded) …is good for the environment (Q15_4)

Daily dairy consumption was calculated using self-reported dietary intake from the FFQ questions. Serving sizes were as follows: milk 200 ml, cheese 40 g, yoghurt 125 g, milk or cream-based desserts 100 g of which 50 g dairy, cheese-based dishes 170 g of which 50 g dairy, based on the WinDiets 2014 database.^(24)^ As the UK does not have fully quantitative guidelines for dairy consumption, the national average of 1.81 dairy portions per day^(3)^ was used to classify participants as either low or high consumers. Where partial postcodes were provided, the decile of the index of multiple deprivation (IMD) and locality (urban/rural) were calculated as the average classification for all postcodes within the given postcode area.^(25–31)^

### Data cleaning and statistical analysis

Submitted data were cleaned (*n* = 108 removed), removing duplicate, implausible, and inaccurate responses using the following predetermined rules: multiple incomplete responses, repeated consecutive ‘*I don’t know*’ or neutral responses, patterned or otherwise repeated responses, and nonsense responses, for example, age >100. The present study analysed responses only from participants who reported consuming dairy in the FFQ; participants who reported following vegan dietary patterns and no dairy consumption were excluded from the present analyses. Respondents (*n* = 17) whose daily milk consumption exceeded 2450 g or 770 g of other dairy products (not milk) were also removed, with cut-off values derived from the maximum (±2 standard deviations) daily intake reported on the NDNS (2016/17–2018/19).

Statistical analyses were performed using RStudio (2023). For continuous variables, a *t*-test or ANOVA was used to examine differences between consumption groups; for categorical data, a chi-squared test was used. Where appropriate, non-parametric tests were used. ANCOVA (or linear models where interactions between consumption level and covariates were identified) were conducted to assess differences in the cumulative attitude scores for each of the four domains, between consumption groups (‘high’ or ‘low’). The initial models included the following covariates: high/low consumption, gender, household income, education attainment, household structure (as number of children in household), urban/rural, dietary pattern (omnivore, pescatarian, vegetarian, other), and subject studied. These were selected based on differences identified in the descriptive statistics analysis and the existing literature on factors affecting attitudes. In the final models, adjustments were made to variables that contributed significantly to the initial model of each domain. Interactions between the primary predictor variable (high/low consumption) and individual covariates were assessed by ANOVA.

Multiple logistic regression for willingness to buy product D (with most attributes linked to nutrition, provenance, sustainability, animal welfare) was conducted; covariates were initially selected in order to assess the influence of respondent demographics (household income, IMD, urban/rural, age, gender, number of children in household), their attitudes and knowledge related to animal welfare (*n* = 3), the environmental impact of dairy production (*n* = 3), the role/importance of dairy in nutrition (*n* = 4) and the influence of price on their purchasing decisions (*n* = 2). Attitude and knowledge variables were based on the previously described cumulative scores. Willingness-to-try scores were based on responses to 5-point Likert questions (Q25); willingness-to-buy scores were factor rankings (1–7) from Q39. Variables that made a significant contribution to the initial model (*p* < 0.05, *n* = 4) were included in the final model: high/low consumption, importance of price in normal purchasing, and cumulative attitude scores for animal welfare and environment.

Where multiple comparisons were made, Benjamini–Hochberg adjustments were made to the accepted *p*-value.

## Results

### Respondent characteristics

The survey received 1908 responses, of which 1801 were valid. In this paper, only individuals who indicated consuming dairy products are included (*n* = 706, data summarised in Table 3). In the final sample, 75% (*n* = 531) of respondents were women, 90% were of white British ethnicity (*n* = 636), and the median age of participants was 46 (IQR 27). The median completion time was 24 min (IQR 15). Based on the FFQ, 53% (*n* = 375) were classified as ‘Low’ dairy consumers and 47% (*n* = 331) as ‘High’ dairy consumers. High consumers had almost twice the odds (OR 1.91, *p* < 0.001) of living in rural areas and studying farming/agriculture (OR 2.14, *p* < 0.001). Additionally, differences in self-reported dietary patterns were identified, specifically a greater proportion of low consumers following vegetarian diets (*p* < 0.001). Regarding health, high consumers had greater odds of reporting being ‘very active’ compared to low consumers (OR 2.41, *p* < 0.001); however, there were no differences in the history of diet-related illnesses, falls, or fractures in the previous 5 years.

**Table 3.** Respondent characteristics stratified by daily dairy consumption Table 3 long description.

	All	Low consumers (<1.81 portions/d)	High consumers (>1.81 portions/d)
***n* (%)**	706	375 (53)	331 (47)
**Gender: female**	531 (75)	291 (78)	240 (73)
**Age, median (IQR)**	46 (27)	46 (27)	46 (28)
**Ethnicity: white British**	636 (90)	330 (88)	306 (92)
**Rural**	367 (52)	167 (45)^a^	200 (60)^b^
**IMD^1^ decile, median (IQR)**	6 (2)	6 (2)	6 (2)
**Household income**			
* Up to £20,000*	104 (15)	58 (15)	46 (14)
* £20,001–£40,000*	190 (27)	89 (24)	101 (31)
* £40,001–£60,000*	137 (19)	78 (21)	59 (18)
* £60,001–£80,000*	77 (11)	42 (11)	35 (11)
* £80,001 and above*	77 (11)	42 (11)	35 (11)
* Prefer not to say*	121 (17)	66 (18)	55 (17)
**Education**			
* Below degree*	243 (34)	114 (30)^a^	129 (39)^b^
* Undergraduate*	267 (38)	143 (38)	124 (37)
* Postgraduate*	180 (25)	111 (30)^a^	69 (21)^b^
* Prefer not to say*	16 (2)	7 (2)	9 (3)
**Subject studied**			
* Nutrition/dietetics*	38 (5)	18 (5)	20 (6)
* Food Sciences/food technology/food chemistry (or similar)*	24 (3)	13 (3)	11 (3)
* Medicine/dentistry/nursing*	51 (7)	25 (7)	26 (8)
* Health sciences (pharmacy, midwifery, other allied health professions)*	43 (6)	25 (7)	18 (5)
* Farming/agriculture*	103 (15)	39 (10)^a^	64 (19)^b^
* Other/prefer not to say*	447 (63)	255 (68)	192 (58)
**Employment**			
* Full-time (>30 h/week)*	343 (49)	174 (46)	169 (51)
* Part-time (<30 h/week)*	143 (20)	82 (22)	61 (18)
* Unemployed/retired*	95 (13)	53 (14)	42 (13)
* Student*	60 (8)	29 (8)	31 (9)
* Prefer not to say*	65 (9)	37 (10)	28 (8)
**Dietary pattern**			
* Omnivore*	546 (77)	275 (73)^a^	271 (82)^b^
* Pescatarian*	15 (2)	11 (3)	4 (1)
* Vegetarian*	86 (12)	64 (17)^a^	22 (7)^b^
* Other*	59 (8)	25 (7)	34 (10)
**Main shopper**	562 (80)	311 (83)	251 (76)
**Main cook**	534 (76)	294 (78)	240 (73)
**Weekly food budget (per person)**			
* <£25*	284 (40)	161 (43)	123 (37)
* £25–£37.50*	214 (30)	117 (31)	97 (29)
* £37.50–£43.75*	54 (8)	27 (7)	27 (8)
* >£43.75*	154 (22)	70 (19)	84 (25)
**Activity**			
* Fairly inactive*	127 (18)	77 (21)	50 (15)
* Moderately active*	499 (71)	272 (73)	227 (69)
* Very active*	78 (11)	26 (7)^a^	52 (16)^b^
**Health**			
* No diet-related illness*	490 (69)	251 (67)	239 (72)
* History of falls*	91 (13)	57 (15)	34 (10)
* History of fractures*	85 (12)	40 (11)	45 (14)

^1^IMD, index of multiple deprivation.

Values are *n* (%) unless otherwise stated. Chi-squared test (categorical data) or *t*-test (continuous data); different superscript letters indicate a significant difference between high- and low-consumption groups at *p* < 0.05 after Benjamini–Hochberg adjustment, raw and adjusted *p*-values presented in SI Table 1.

Superscript letters are used to indicate where differences were significant at p<.05, as per footnote, with exact P values are provided in full in SI Table 1.

### Purchasing and consumption behaviours

**Figure 1.**
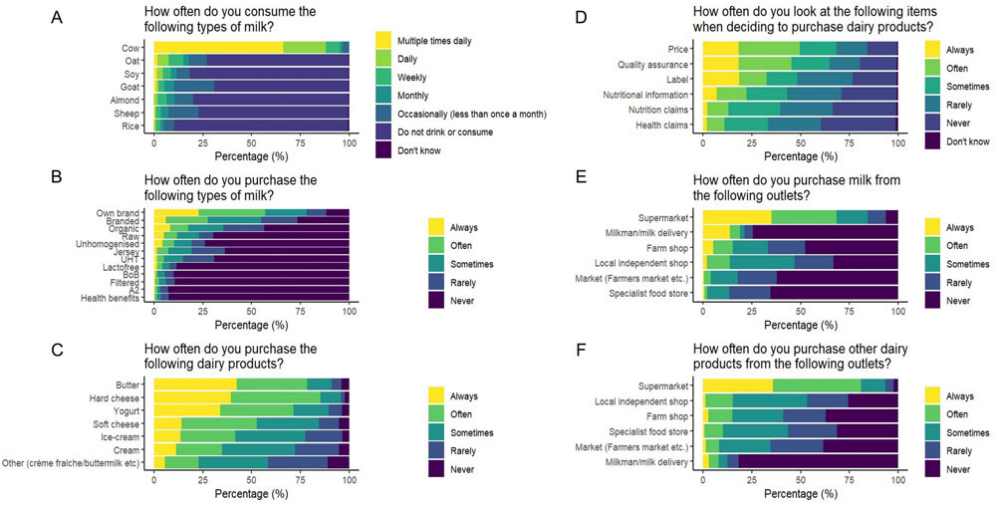
Figure 1 long description. Dairy consumption and purchasing habits of survey respondents. (A) Consumption frequency of different types of milk, including plant-based alternatives. (B) Milk purchasing habits – milk type. (C) Frequency of purchasing other dairy products. (D) Considerations for habitual purchasing of dairy products. (E and F) Purchasing habits – shop type.

Supermarket own-brand milk was most frequently purchased (Figure 1B), with branded products accounting for approximately 25% of purchases. Fewer than 20% of participants regularly bought organic, raw, or unhomogenised milk. As such, milk was predominantly purchased from supermarkets (75% often/always), whilst a small proportion of respondents received milk by doorstep delivery or purchased from specialist retailers (Figure 1E). Similar patterns were seen for the other dairy products (Figure 1F). Generally, price was the most important factor determining purchasing behaviour, followed by the presence of a quality assurance brand (Figure 1D). Less than a quarter (22%) of respondents indicated that on-pack nutrition information was important. In comparison, nutrition and health claims were considered very infrequently (59% and 65%, respectively).

Median daily consumption was 201 mL (IQR 95–380 mL) for milk and 129 g (IQR 74–185 g) for other dairy products, giving an overall median of 1.7 daily portions of dairy (IQR 1.0–2.8). Milk was most often consumed with tea and coffee, but milk with breakfast cereal made the greatest contribution to overall consumption.

### Knowledge of dairy production and nutrition

Cumulative scores for knowledge ranged from 17% to 96% of the maximum possible score. There was no difference in knowledge between high and low-consumer groups (*p* = 0.061). Across all respondents, knowledge concerning animal husbandry and farming practices had an average score of 72%. However, within this section, two (out of ten) questions requiring high industry knowledge (average number of days cows spend outside and annual milk production volume) were poorly answered (60% incorrect and 70% ‘*I don’t know*’). In addition, responses to why manure is a concern for climate change were split between two answers: 49% of respondents correctly identified that polluting rivers and waterways are a concern, whilst 41% answered that decomposition releases nitrous oxide. For nutrition, the mean score was 71%; most participants correctly matched nutrients to their physiological functions (83–99% correct), but specific knowledge concerning milk’s nutritional qualities was lower. Milk is considered a good source of calcium (98% correct), vitamin B_12_ (62% correct), and iodine (54% correct). However, many participants incorrectly considered milk to be a good source of vitamin D (56%), omega-3 fatty acids (41%), omega-6 fatty acids (35%), iron (34%), and folic acid (34%).

### Attitudes towards dairy

**Figure 2.**
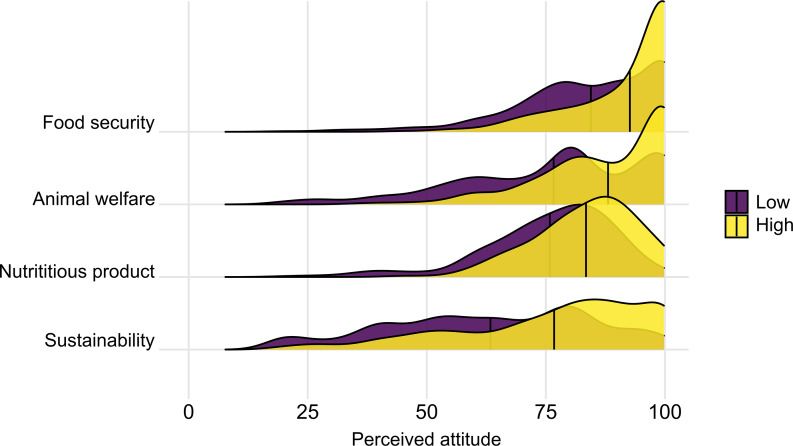
Figure 2 long description. Cumulative scores, shown as percentages, calculated using responses to Likert-type questions (e.g. 1 – strongly disagree; 5 – strongly agree) relating to the four domains: food security, animal welfare, dairy as nutritious products, and sustainability of dairy production). Higher scores indicate more positive attitudes. Where necessary, responses were reverse coded, for example, Q15.2 ‘The dairy industry in the UK is a major source of nitrogen emissions’, ‘strongly disagree’ scored 5. Responses stratified by consumption (high ≥ 1.81 portions/d; low < 1.81 portions/d). Higher scores indicate a more positive attitude.

Consumption level was associated with higher scores across all domains (*p* < 0.001, Figure 2), and adjusting for sociodemographic and lifestyle covariates did not alter this, except for attitude towards animal welfare (*p* = 0.17). High dairy consumption was associated with cumulative scores 6–8% higher compared to low dairy consumption, regardless of adjustment for demographic covariates (SI Tables 2 & 3). Attitudes towards animal welfare did not differ between high- and low-consumption groups (*p* = 0.218); however, we identified an interaction between gender and consumption. Among women, higher dairy consumption was associated with higher attitude scores for animal welfare compared to women in the lower consumption group (+12%, *p* = 0.022). In contrast, there was no difference among men, regardless of consumption level.

**Figure 3.**
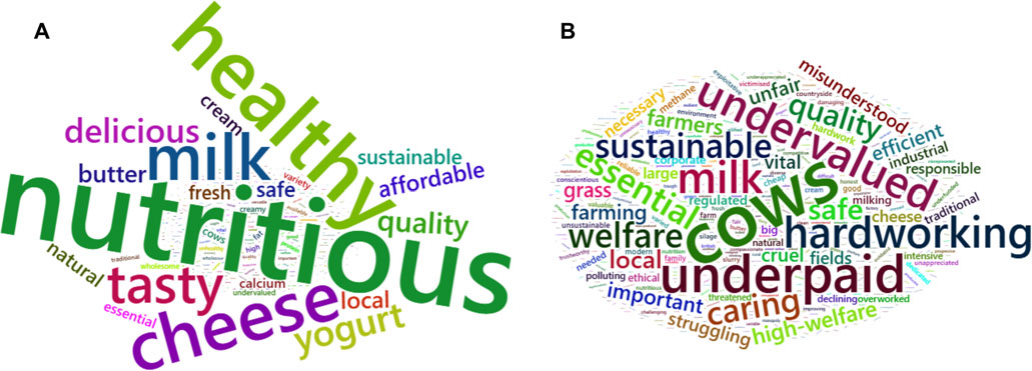
Figure 3 long description. Words used by consumers to describe (A) Dairy products (B) The dairy industry.

Among all respondents, the overall sentiment towards dairy products was positive (*n* = 1983 words, summed AFINN score: 506, Figure 3). Within the high consumer group, there was a sentiment that the dairy industry is important to UK food security, with a median score of 100 (IQR 19), compared to 81 (IQR 36) for low consumers. We found that lower consumption was associated with greater scepticism regarding sustainability and the dairy industry, as indicated by a median score that was 25% lower than that of the high consumer group. Respondents emphasised the nutritional benefits of dairy products and hedonic properties, for example, ‘*tasty*’ and ‘*delicious*’ (Figure 3A). Support for dairy farmers and the industry was highlighted through words such as ‘*hardworking*’, ‘*underpaid*’, ‘*important*’ and ‘*necessary*’, whilst others focused on animal welfare, using descriptors like ‘*caring*’ and ‘*high welfare*’ (Figure 3B). Analysis of free text responses identified conflicting sentiments about industry practices; several comments indicated disparities between small-scale farms and larger, more industrialised herds in terms of ethics, sustainability, and nutritional qualities of the milk. There were 83 comments relating to unfair treatment of farmers, especially in economic terms, and concern that low farmgate prices may be detrimental to on-farm practices. A theme identified among high consumers’ comments was that UK farmers care for their animals and maintain very high welfare standards, whereas low consumers expressed greater concern about industry practices. These comments were often qualified by the notion that welfare and environmental consequences vary greatly across production systems; in particular, respondents associated small-scale production with higher animal welfare and dairy production with positive contributions to local landscapes and biodiversity. Several responses, particularly from high consumers, compared the British dairy industry with the rest of the world, suggesting that its welfare standards are superior. However, others raised concerns about the treatment of bull calves, the use of artificial insemination, and the separation of cows and calves. Information and knowledge emerged as another theme, in particular: (mis)information spread through mainstream and social media platforms, a need for more information about production on packaging, and a personal lack of knowledge about the industry and its impacts. Comments regarding methane ranged from acknowledging the emissions associated with dairy production to suggestions that the industry is used as a scapegoat for more damaging industries, such as transport. Other comments suggested that the impacts are overstated because of the relatively short half-life of methane compared to other greenhouse gases. Additionally, some respondents compared dairy to PBA with respect to environmental consequences (e.g. food miles and monocultures) and nutritional qualities.

### Willingness to buy improved dairy products

**Figure 4.**
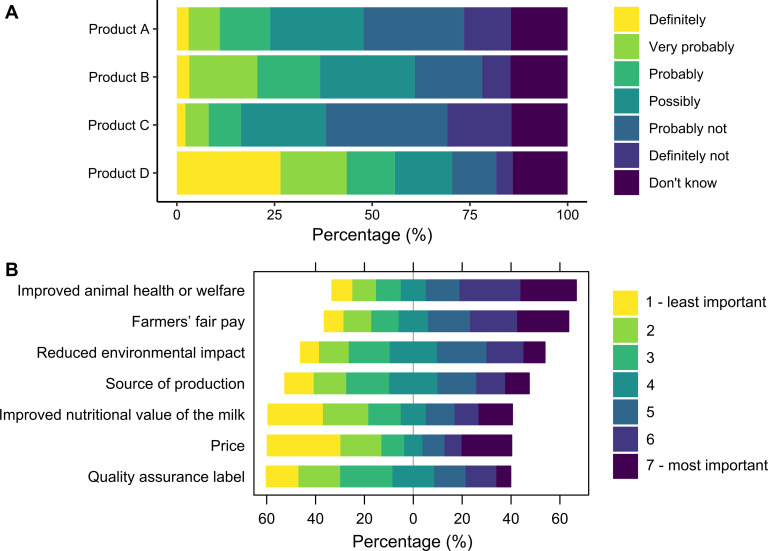
Figure 4 long description. Responses to full profile conjoint analysis, (A) Participant likelihood to purchase milk products with different attributes presented on their packaging. (B) Ranked importance (1–7) of factors affecting the likelihood of buying the different milk products.

When presented with four milk products bearing different claims about production and nutrition (Table 1), respondents largely preferred Product D, which inferred that the product had natural nutritional enrichment, improved welfare and environmental claims, a clear statement of production location, and that farmers were receiving a fair price. Conversely, Product C, which, much like standard supermarket milk, bore only a standard quality assurance label and was priced 30% cheaper, was least favoured (Figure 4A). Product B was slightly more favoured than product A, indicating that improved welfare and sustainability are more valued attributes than local production and that there is a preference for natural nutritional enrichment rather than fortification during processing. We found that participants perceived welfare to be the most important factor affecting purchasing preferences (ranked as most or second most important by 50% of participants), followed by farmers’ fair pay (most or second most important for 36%). In contrast, price, improved nutrition, and novel quality assurance were ranked least important (ranked least or second least important by 29–49% participants, Figure 4B). Due to the survey design, the relative weights of these individual attributes could not be quantified.

In the initial multivariable logistic model (SI Table 4), we identified four variables associated with a preference for Product D and included them in the final model (SI Table 5). These were consumption level (high/low), reported attitudes towards animal welfare and the environment, and the role of price in self-reported purchasing behaviour. Lower dairy consumption was associated with an increased likelihood of expressing a preference for Product D (OR 1.61, 95% CI 1.22; 1.67, *p* < 0.001). For participants who reported greater importance of price in their habitual purchasing behaviours, we identified an inverse association with a preference for Product D (OR 0.74, 95% CI 0.59; 0.91, *p* = 0.005). Greater cumulative scores for attitudes towards animal welfare were associated with a small increase in preference for Product D (OR 1.03, 95% CI 1.01; 1.05, *p* = 0.007), whilst attitudes towards the environment were associated with a slight reduction in preference for Product D (OR 0.97, 95% CI 0.96; 0.99, *p* = 0.001).

## Discussion

Our findings align with the growing body of evidence indicating that consumers are becoming increasingly aware of the ethical and environmental implications of their dietary choices. However, a significant gap exists between attitudes, intentions, and behaviours. Whilst many consumers express concerns about dairy consumption, most participants do not purchase milk produced in line with their stated values; instead, they choose products predominantly based on price. Our findings corroborate with those of the European Consumer Observatory, which found that 71% of consumers intend to live sustainably, but less than half agree that they currently consider sustainability in their diet.^(32)^

For most foods, taste is considered the most important factor in purchasing decisions^(33,34)^; however, in the present study, we found that price had the greatest importance when habitually purchasing milk, despite 56% of respondents indicating that price had a low influence on preference in the full-profile conjoint analysis. This finding highlights the difference between habitual decision-making and deliberate decision-making in a choice experiment. Additionally, the importance of the different factors in the willingness-to-buy element was based on participants ranking each factor rather than being elucidated through trade-off situations, and may therefore be subject to response bias. Milk is primarily consumed as part of other drinks or foods rather than on its own (e.g. in tea or coffee or on cereal), so its taste may be less important than that of other foods. Alternatively, some consumers may have different preferences for milk type depending on the host food, for example, preferring oat milk in coffee but cows’ milk with cereal. We found that 22% of individuals who consumed cows’ milk at least weekly also regularly consumed a PBA. Recent studies have highlighted that, due to taste, ethics, and perceived healthfulness, many younger coffee consumers prefer PBA in coffee, despite cafes regularly imposing additional charges.^(35–37)^ The development of barista-blend products, designed to have desirable qualities for speciality coffee, has enabled the expansion of PBA into coffee shops and coffee culture.^(36)^ A similar opportunity exists for innovation within the dairy sector, especially when combined with consumer priorities for improved welfare, sustainability, and increasing desire for natural products.^(38)^

Milk is a ubiquitous, fresh product which is generally purchased multiple times a week.^(39,40)^ The high purchasing frequency means that behaviours are habitual,^(41)^ reducing variation in product choice and the influence of packaging and branding. Supermarket choice architecture directs customers towards the supermarket’s own-brand products, whilst alternative products are generally placed out of eyeline, requiring customers to actively search for them.^(42)^ In contrast, other dairy products, such as cheese, have greater variability and are more likely to be consumed on their own, increasing the relative importance of taste compared to price.^(43)^ It should be noted that during the data collection period, milk prices increased from 34.51 pence per litre (ppl) in November 2021 to 38.57 ppl in April 2022, an increase of nearly 10 pence from 12 months earlier.^(44)^ Whilst farmgate milk prices often show considerable volatility, these are seldom reflected in supermarket milk prices, which are instead largely influenced by inflation. During the data collection period, inflation rapidly increased, putting economic strain on household budgets and increasing sales of supermarket own-branded products,^(45)^ hence providing a potential explanation for the relative importance of price in the present study.

The discrepancy between stated values and purchasing habits indicates an ‘intention–behaviour gap’, in which an individual does not behave in the way that would be predicted by their beliefs and values, under the TPB.^(46)^ Intention–behaviour gaps have been widely reported for sustainable consumption, even among ethically minded consumers.^(47)^ Beyond economic constraints, the intention–behaviour gap in milk purchasing may stem from cognitive dissonance about industry practices; however, this would benefit from further exploration. Ioannidou et al.^(48)^ found that dairy consumers adopt a variety of cognitive dissonance strategies to rationalise their consumption, which may be exacerbated by poor knowledge of industry practices. Although animal welfare was a pertinent theme in the qualitative analysis, consumers’ perceptions were largely positive, with 73–90% agreement to statements relating to animal welfare. The extent of cognitive dissonance may vary between different dairy products; for example, on average, vegetarians consume 24% less liquid milk but 74% more cheese than meat-eaters,^(49)^ possibly explained by cheese being eaten for pleasure, being considered a good source of protein, or the additional processing increasing dissociation from its production, compared to milk.^(50)^ Despite a mean nutrition knowledge score of 71%, several misconceptions about micronutrients persisted, potentially due to the overall perception that milk is a nutritious product. Public awareness of the micronutrients provided by milk could be improved through clear nutritional labelling; however, its impact may be limited as nutrition was seldom reported as an important determinant of purchasing behaviour.

Existing research overwhelmingly indicates that sustainability is rarely a priority for dietary choices.^(32,51,52)^ Likely explanations for this include a lack of knowledge about the extent of the environmental impact of dairy^(53)^ and the perception that individual changes have little effect or entail too great a personal cost (i.e. taste, price).^(52)^ Our findings highlight that dairy products are considered healthy and nutritious; thus, consumers may be unwilling to reduce consumption due to healthfulness.^(54)^ Furthermore, we found that more favourable attitudes towards the impact of dairy on the environment were associated with a reduction in the likelihood of expressing a preference for Product D. This may be explained by a belief that dairy production is ‘*natural*’ and ‘*increases biodiversity*’. Additionally, perceptions and definitions of sustainability often differ among stakeholders^(18)^ and, in the context of dairy, may be conflated with terms such as organic.^(55,56)^

Many respondents expressed favourable attitudes towards dairy farmers, particularly that they care for their animals, but are poorly treated by other actors within the food system. Similarly, the EU Trust Report^(32)^ found that farmers were the most trusted actors in the food system, according to citizens. However, it should be noted that attitudes vary substantially based on production systems. In the present study, several respondents commented on differences between pasture-fed, organic, and calf-and-cow systems compared to zero-grazing and intensive production systems. This sentiment aligns with Shortall,^(57)^ who found that intensive production systems were associated with reduced animal welfare and increased environmental impact, whilst grass-based production systems are perceived as more natural. A German taste-test study^(56)^ found that consumer preferences were highly influenced by packaging and credence claims, but taste preferences varied when tasting either blind or nonblind. In blinded tests, participants could not distinguish between grass-fed, organic, and standard milk, but nonblinded participants showed a significant preference for organic milk. Other studies have suggested that people consume less meat if it is described as ‘*factory farmed*’ compared to ‘*humanely farmed*’ due to greater emotional engagement with the animals,^(58)^ whilst Scott et al.^(59)^ found that people were able to disconnect consumption habits from animal suffering. Some respondents in the present study felt that negative media portrayal of the dairy industry has led to an exaggeration of the environmental impacts of consumption, that dairy is of cultural importance, and that changes to the industry should be led by national policies and legislation rather than individual action. These perspectives may explain the differences in animal welfare attitude scores observed among women in the high-consumption group compared to the low-consumption group. Women in the high-consumption group had the highest overall scores for animal welfare (90%), indicating that they had a stronger agreement with statements such as ‘*dairy farmers in the UK care for their animals*’, whilst the women in the low-consumption group had the lowest overall scores (78%).

A recurring sentiment that British dairy products are produced to higher ethical, environmental, and quality standards than those in the EU was present throughout many responses; however, justification was seldom provided. These comments were particularly prevalent among individuals residing in rural areas, who are more likely to be involved in or have a greater affinity for agriculture.^(60)^ Additionally, these respondents had higher dairy product intake than people living in urban areas, concurring with the UK Household Food Survey,^(3)^ which reported that rural dwellers consume approximately 200 mL more milk and other dairy products per capita per week. Whilst consumers reported that seeing quality assurance branding was an important factor when purchasing dairy products, the presence of quality assurance branding had the lowest influence on the likelihood to buy section. This corroborates findings of Food Standards Scotland^(51)^: cost was most important, followed by taste, whilst provenance and environmental impact were the least important. Purchasing products with a quality assurance logo may make consumers feel they are ‘*doing the right thing*’; however, despite high recognition of the Red Tractor logo, a UK survey indicated that 64% of consumers need clarification on what the scheme means.^(61)^ The presence of the Red Tractor logo may lead consumers to believe the product has been produced to very high trade standards; however, a comparison of animal welfare standards across different quality assurance brands found that Red Tractor certification only ensures that the minimum legislative standards are adhered to.^(62)^ Furthermore, whilst consumers may intend to purchase products with specific traits, personal values are often overridden, particularly for price-conscious consumers, where assurance brands carry a price premium.^(63)^

Whilst many respondents commented on the polluting effects of methane and manure, consumers generally did not feel that the industry has a negative impact on the environment or is a major source of greenhouse gas emissions. In some cases, differences in greenhouse gas emissions’ half-lives and emissions from other industries were offered as an explanation. Studies indicate a need for greater understanding of how dietary choices affect the environment.^(18,52,64)^ Additionally, cognitive dissonance may be perpetuated by perceptions of tradition and culture surrounding the role of farming and cows in Britain’s natural landscape.

### Limitations

The scope and length (median completion time 24 min) of the survey meant we could not fully operationalise all TPB elements, such as subjective norms, without incurring a substantial increase in participant burden. Although the survey had a good response rate, due to the nature of the topic, the respondent characteristics showed that non-consumers (excluded from analysis) and individuals with links to agriculture were overrepresented, highlighting that our findings should not be generalised to the UK population as a whole. The overrepresentation of people with links to farming has likely introduced a pro-farming bias, as indicated by the qualitative data. Additionally, women were overrepresented, whilst there was an underrepresentation of non-white ethnicities. As such, we recommend that future studies consider stratified recruitment strategies to ensure a representative sample. The question that contributed most to the qualitative data was optional, so responses from individuals with stronger feelings towards the industry likely skewed the data. The likelihood-to-purchase question was not designed as a discrete-choice experiment, limiting our ability to assess consumer preference for each product quantitatively. Data collection (November 2021–April 2022) took place around the same time as the spread of the Omicron COVID-19 variant in the UK (first detected 27^th^ November 2021). Whilst many people were isolating during this period, there were no widespread restrictions beyond social distancing and wearing of face covering in public spaces. Schools, restaurants, and supermarkets were open throughout the period. Consumer trends data indicate that expenditure on the dairy, eggs, and cheese category did not differ substantially from pre-pandemic levels.^(65)^ Card spending data showed that expenditure in pubs, restaurants, and fast food outlets was 7% lower in December 2021 compared to December 2019; however, there was a 7% increase in spending on food and drink (from supermarkets, convenience stores, etc.), indicating that whilst there were changes in consumer behaviours we cannot quantify the impact on dairy products specifically. Finally, the data collection period did, however, coincide with a period of substantial inflation in the UK, which may have influenced the role of price in purchasing behaviours, and willingness to pay more for improved products, in turn implicating the role of other factors such as ethical or environmental concerns.

## Conclusion

We found that consumers valued dairy’s healthfulness and taste alongside ethics, provenance, and sustainability, yet prioritised price in purchasing behaviours. Participants, particularly those in the high-consumption group, had positive attitudes towards dairy farmers in terms of ethical standards within the industry, which may diminish their willingness to pay for an alternative dairy product within the same category. In contrast, concerned consumers may choose to purchase PBA. Sustainability was otherwise rarely a priority determinant of purchasing behaviours and was further compounded by several misconceptions regarding environmental impacts of dairy production. These findings emphasise consumers’ substantial cognitive dissonance towards ethical and sustainable diets. Whilst there is a complex nexus between absolute, perceived, and understood knowledge, clear and transparent labelling, such as an easily identifiable quality assurance brand highlighting improved production standards, could help consumers make better-informed purchasing decisions. Additionally, policies and legislation are necessary to support farmers and consumers in producing and purchasing more sustainable products.

## Supporting information

10.1017/jns.2026.10109.sm001 Bracey et al. supplementary material 1 Bracey et al. supplementary material

10.1017/jns.2026.10109.sm002 Bracey et al. supplementary material 2 Bracey et al. supplementary material

## Supplementary material

The supplementary material for this article can be found at https://doi.org/10.1017/jns.2026.10109.

## Table 1. Long description

The table compares four milk products, labeled A, B, C, and D, across several attributes. Product A is fortified with iodine, vitamin D, selenium, and omega-3 during processing and is produced locally or regionally, with a cost of 80 pence per litre. Product B cows are fed a diet high in iodine, vitamin D, selenium, and omega-3, improving animal health and welfare and reducing environmental impact, costing 90 pence per litre. Product C also focuses on a high diet for cows, improving animal health and welfare, with a cost of 70 pence per litre. Product D includes all the attributes of Product C, states production and processing locations, and is a novel brand, costing 1 pound per litre. The table highlights differences in fortification methods, animal welfare, sustainability, origin, quality assurance, fair trade, and cost per litre among the products.

Navigate back to Table 1..

## Table 2. Long description

The table presents variables contributing to attitude scoring in the dairy industry, focusing on four key domains: environment/sustainability, animal welfare, nutrition, and UK food security. It includes statements related to each domain, such as the dairy industry’s impact on the environment, animal welfare practices, the nutritional value of milk, and the importance of dairy production for UK food security. The table has four rows and three columns, with column headers indicating the domain and row labels listing specific statements. Notable trends include higher scores indicating positive perceptions of the dairy industry’s environmental impact, favorable attitudes towards animal welfare, the belief that milk is nutritious, and the importance of dairy production for food security. The data type involves Likert-scale responses scored from 1 to 5 points, with some responses reverse coded to align with the scoring. The table aims to provide a comprehensive overview of public attitudes towards the dairy industry based on various factors.

Navigate back to Table 2..

## Table 3. Long description

The table presents data on 706 respondents who consume dairy products, categorized into low consumers (less than 1.81 portions per day) and high consumers (more than 1.81 portions per day). It includes details on gender, age, ethnicity, rural living, household income, education, subject studied, employment status, dietary patterns, weekly food budget, activity level, and health status. Key trends include a higher percentage of women (75%), white British ethnicity (90%), and a median age of 46. High consumers are more likely to live in rural areas and study farming/agriculture. Notable differences in dietary patterns and activity levels are also highlighted.

Navigate back to Table 3..

## Figure 1. Long description

The image contains six bar graphs depicting various aspects of dairy consumption and purchasing habits. Graph A shows the frequency of consuming different types of milk, including cow, oat, soy, goat, almond, sheep, and rice milk. Graph B illustrates the purchasing habits of different types of milk, such as own brand, branded, organic, and others. Graph C presents the frequency of purchasing other dairy products like butter, hard cheese, yogurt, soft cheese, ice cream, and cream. Graph D highlights the considerations for habitual purchasing of dairy products, including price, quality assurance, label, nutritional information, nutrition claims, and health claims. Graphs E and F depict the purchasing habits from different outlets, such as supermarkets, milkman/milk delivery, local independent shops, farm shops, markets, and specialist food stores for milk and other dairy products respectively. Each graph uses a color-coded scale to represent different frequencies or considerations.

Navigate back to Figure 1..

## Figure 2. Long description

The image consists of two word clouds labeled A and B. Word cloud A uses various colors and sizes to depict positive terms associated with dairy products, such as ‘healthy,’ ‘nutritious,’ ‘milk,’ ‘tasty,’ and ‘cheese.’ Word cloud B highlights terms related to the dairy industry, including ‘undervalued,’ ‘hardworking,’ ‘cows,’ ‘sustainable,’ and ‘underpaid.’ The words are arranged in a circular pattern, with larger fonts emphasizing more frequently used terms. The visual representation contrasts the positive consumer perception of dairy products with the more critical view of the dairy industry.

Navigate back to Figure 2..

## Figure 3. Long description

The line graph presents attitudes towards the dairy industry, segmented into four domains: food security, animal welfare, nutritious products, and sustainability. The x-axis represents perceived attitude on a scale from 0 to 100, while the y-axis lists the four domains. Two data lines, labeled ‘Low’ and ‘High,’ indicate responses stratified by consumption levels, with ‘Low’ representing less than 1.81 portions per day and ‘High’ representing 1.81 portions per day or more. The ‘Food security’ domain shows a gradual increase in positive attitudes, peaking sharply at the high end. ‘Animal welfare’ exhibits a steady rise with a notable peak towards the high end. ‘Nutritious product’ follows a similar trend, with a significant increase in positive attitudes. ‘Sustainability’ shows a more gradual and consistent rise across the spectrum. All values are approximated.

Navigate back to Figure 3..

## Figure 4. Long description

The image contains two horizontal bar charts. The first chart, labeled A, shows the likelihood of purchasing different milk products with various attributes. The x-axis represents the percentage of participants, ranging from 0 to 100 percent. The y-axis lists four products: Product A, Product B, Product C, and Product D. Each bar is segmented into different colors representing responses: Definitely, Very probably, Probably, Possibly, Probably not, Definitely not, and Don’t know. The second chart, labeled B, shows the ranked importance of factors influencing the likelihood of buying different milk products. The x-axis represents the percentage of participants, ranging from 0 to 60 percent. The y-axis lists seven factors: Improved animal health or welfare, Farmers’ fair pay, Reduced environmental impact, Source of production, Improved nutritional value of the milk, Price, and Quality assurance label. Each bar is segmented into different colors representing the importance ranking from 1 (least important) to 7 (most important).

Navigate back to Figure 4..

## References

[1] Davoodi SH , Shahbazi R , Esmaeili S , et al. Health-related aspects of milk proteins. Iran J Pharm Res. 2016;15:573–591. PMC5149046 27980594

[2] Alothman M , Hogan SA , Hennessy D , et al. The “Grass-Fed” milk story: understanding the impact of pasture feeding on the composition and quality of bovine milk. Foods. 2019;8:350. 31426489 10.3390/foods8080350 PMC6723057

[3] DEFRA. Family food datasets. 2023. Accessed April 2026. https://www.gov.uk/government/collections/family-food-statistics.

[4] Statista. Supermarket brands of milk ranked by number of consumers in Great Britain in 2023. 2024. Accessed April 2026. https://www.statista.com/statistics/307010/leading-supermarket-brands-of-milk-uk/.

[5] Pellegrino L , Marangoni F , Muscogiuri G , et al. Vitamin D fortification of consumption cow’s milk: health, nutritional and technological aspects. A multidisciplinary lecture of the recent scientific evidence. Molecules. 2021;26:5289. 34500722 10.3390/molecules26175289 PMC8434398

[6] Walker GP , Dunshea FR , Doyle PT. Effects of nutrition and management on the production and composition of milk fat and protein: a review. Aust J Agr Res. 2004;55:1009–1028.

[7] Kliem KE , Shingfield KJ. Manipulation of milk fatty acid composition in lactating cows: opportunities and challenges. Eur J Lipid Sci Tech. 2016;118:1661–1683.

[8] Moyersoen I , Lachat C , Cuypers K , et al. Do current fortification and supplementation programs assure adequate intake of fat-soluble vitamins in Belgian infants, toddlers, pregnant women, and lactating women? Nutrients. 2018;10:223. 29462926 10.3390/nu10020223 PMC5852799

[9] Niedermaier T , Gredner T , Kuznia S , et al. Vitamin D food fortification in European countries: the underused potential to prevent cancer deaths. Eur J Epidemiol. 2022;37:309–320. 35524028 10.1007/s10654-022-00867-4 PMC9187526

[10] European Commission. Food-based dietary guidelines recommendations for milk and dairy products. 2024. Accessed April 2026. https://knowledge4policy.ec.europa.eu/health-promotion-knowledge-gateway/food-based-dietary-guidelines-europe-table-7_en.

[11] Public Health England. National Diet and Nutrition Survey Rolling programme Years 9 to 11 (2016 to 2017 and 2018 to 2019) NDNS: results from years 9 to 11 (2016 to 2017 and 2018 to 2019) - GOV.UK. 2020.

[12] Dineva M , Rayman MP , Bath SC. Iodine status of consumers of milk-alternative drinks v. cows’ milk: data from the UK National Diet and Nutrition Survey. Br J Nutr. 2021;126:28–36. 32993817 10.1017/S0007114520003876

[13] Witard OC , Bath SC , Dineva M , et al. Dairy as a source of iodine and protein in the UK: implications for human health across the life course, and future policy and research. Front Nutr. 2022;9:800559. 35223949 10.3389/fnut.2022.800559 PMC8866650

[14] Agriculture and Horticulture Development Board. Reputation factors outweigh cost as a reason for reduction of dairy. 2023. Accessed April 2026. https://ahdb.org.uk/news/consumer-insight-reputation-factors-outweigh-cost.

[15] Agriculture and Horticulture Development Board. Trust in British agriculture and consumer perceptions on the environment. 2023. Accessed April 2026. https://ahdb.org.uk/news/consumer-insight-trust-in-british-agriculture-and-consumer-perceptions-on-the-environment.

[16] Regan Á. , Kenny U. What do the public want to know about farming and why? Findings from a farmer-initiated public consultation exercise in Ireland. Sustainability. 2022;14:5391.

[17] Hartmann C , Lazzarini G , Funk A , et al. Measuring consumers knowledge of the environmental impact of foods. Appetite. 2021;167:105622. 34363900 10.1016/j.appet.2021.105622

[18] Schiano AN , Harwood WS , Gerard PD , et al. Consumer perception of the sustainability of dairy products and plant-based dairy alternatives. J Dairy Sci. 2020;103:11228–11243. 33069414 10.3168/jds.2020-18406

[19] Office for National Statistics. Population estimates. 2018. Accessed April 2026. https://www.ons.gov.uk/peoplepopulationandcommunity/populationandmigration/populationestimates.

[20] Ajzen I. The theory of planned behavior. Organ Behav Hum Dec. 1991;50:179–211.

[21] Sun W. Toward a theory of ethical consumer intention formation: re-extending the theory of planned behavior. AMS Review. 2020;10:260–278.

[22] Bingham SA , Welch AA , McTaggart A , et al. Nutritional methods in the European prospective investigation of cancer in Norfolk. Public Health Nutr. 2001;4:847–858. 11415493 10.1079/phn2000102

[23] Neilsen F. A new ANEW: Evaluation of a word list for sentiment analysis in microblogs. In Proceedings of the ESWC2011 Workshop on Making Sense of Microposts: Big things come in small packages 718. CEUR Workshop Proceedings: Informatics and Mathematical Modelling, Technical University of Denmark.2011.

[24] WinDiets. In Nutr Food Sci, 1.0.4900 ed., vol. 32: Emerald Group Publishing Limited.

[25] Scottish Government. Scottish index of multiple deprivation 2020. 2020. Accessed April 2026. https://www.gov.scot/collections/scottish-index-of-multiple-deprivation-2020/.

[26] Ministry of Housing Communities & Local Government. English indices of deprivation 2019. 2019. Accessed April 2026. https://www.gov.uk/government/statistics/english-indices-of-deprivation-2019.

[27] NISRA. Northern Ireland multiple deprivation measure 2017 (NIMDM2017). 2017. Accessed April 2026. https://www.nisra.gov.uk/statistics/deprivation/northern-ireland-multiple-deprivation-measure-2017-nimdm2017.

[28] Cymru L. Welsh index of multiple deprivation. 2022. Accessed April 2026. https://www.gov.wales/welsh-index-multiple-deprivation.

[29] DEFRA. Rural urban classification. 2016. Accessed April 2026. https://www.gov.uk/government/collections/rural-urban-classification.

[30] Scottish Government. Scottish Government urban rural classification 2020. 2022. Accessed April 2026. https://www.gov.scot/publications/scottish-government-urban-rural-classification-2022/.

[31] NISRA. Urban - rural classification. 2015. Accessed April 2026. https://www.nisra.gov.uk/support/geography/urban-rural-classification.

[32] EIT Food Consumer Observatory. Trust Report. 2024. Accessed April 2026. https://www.eitfood.eu/reports/trust-report-2024.

[33] European Commissions. Special Eurobarometer 505: Making our food fit for the future - Citizens’ expectations. 2020. Accessed April 2026. https://data.europa.eu/data/datasets/s2241_505_eng?locale=en.

[34] Torán-Pereg P , Mora M , Thomsen M , et al. Understanding food sustainability from a consumer perspective: a cross cultural exploration. Int J Gastron Food Sci. 2023;31:100646.

[35] Adamczyk D , Jaworska D , Affeltowicz D , et al. Plant-based dairy alternatives: consumers perceptions, motivations, and barriers-results from a qualitative study in Poland, Germany, and France. Nutrients. 2022;14:2171. 35631311 10.3390/nu14102171 PMC9147774

[36] Halabi N , Hristova V , Vlaev I. Milking the alternatives: understanding coffee consumers preferences for non-dairy milk. Behav Sci (Basel). 2024;14:569. 39062392 10.3390/bs14070569 PMC11273792

[37] Gupta A , Keast R , Liem DG , et al. Barista-quality plant-based milk for coffee: a comprehensive review of sensory and physicochemical characteristics. Beverages. 2025;11:24.

[38] Kershaw J , Nolden A , Ellinger L , et al. Consumers’ perceptions of plant-based alternatives relative to the foods they directly imitate. Food Qual Prefer. 2025;129:105519.

[39] Merlino VM , Massaglia S , Borra D , et al. Which factors drive consumer decisions during milk purchase? New individuals’ profiles considering fresh pasteurized and UHT treated milk. Foods. 2021;11:77. 35010206 10.3390/foods11010077 PMC8750682

[40] Bytyqi N , Muji S , Rexhepi A. Consumer behavior for milk and dairy products as daily consumption products in every household—The case of Kosovo. Open J Bus Manag. 2020;08:997–1003.

[41] Mariusz G. Consumer determinants of purchasing decisions on the dairy products market. Eur Res Stud J. 2021;24:981–992.

[42] Adukia S. 2023. Competitive advantages of choice architecture in marketing compared to traditional marketing methods: A guide for multinational corporations. NHSJS Reports.

[43] Streletskaya NA , Maruyama S , Queisser S , et al. How information leads consumers to select specialty foods when tasting is not an option. Food Qual Prefer. 2023;105:104769.

[44] Agriculture and Horticulture Development Board. 2024. UK Farmgate all milk prices. Accessed August 20, 2024. https://ahdb.org.uk/dairy/uk-farmgate-milk-prices.

[45] Kantar. Brand brinkmanship - the quiet transformation. 2023. Accessed April 2026. https://www.kantar.com/uki/campaigns/2023-wp-brand-brinksmanship.

[46] Fink L , Strassner C , Ploeger A. Exploring external factors affecting the Intention-behavior gap when trying to adopt a sustainable diet: a think aloud study. Front Nutr. 2021;8:511412. 33681270 10.3389/fnut.2021.511412 PMC7933023

[47] Carrington MJ , Neville BA , Whitwell GJ. Why ethical consumers don’t walk their talk: towards a framework for understanding the gap between the ethical purchase intentions and actual buying behaviour of ethically minded consumers. J Bus Ethics. 2010;97:139–158.

[48] Ioannidou M , Lesk V , Stewart-Knox B , et al. Feeling morally troubled about meat, dairy, egg, and fish consumption: dissonance reduction strategies among different dietary groups. Appetite. 2023;190:107024. 37673128 10.1016/j.appet.2023.107024

[49] Bradbury KE , Tong TYN , Key TJ. Dietary intake of high-protein foods and other major foods in meat-eaters, poultry-eaters, fish-eaters, vegetarians, and vegans in UK Biobank. Nutrients. 2017;9:1317. 29207491 10.3390/nu9121317 PMC5748767

[50] Docherty D , Jasper C. The cheese paradox: how do vegetarians justify consuming non-meat animal products? Appetite. 2023;188:106976. 37454766 10.1016/j.appet.2023.106976

[51] Food Standards Scotland. Consumer attitudes towards the diet and food environment in Scotland. 2023. Accessed April 2026. https://www.foodstandards.gov.scot/sites/default/files/migration/downloads/Consumer_attitudes_towards_the_diet_and_food_environment_in_Scotland_research_report_-_June_2023.pdf.

[52] Food Standards Agency. 2021. A rapid review of the evidence on the factors underpinning the consumption of meat and dairy among the general public. A rapid review of the evidence on the factors underpinning the consumption of meat and dairy among the general public|food standards agency. Accessed April 2026.

[53] Food Standards Agency. Healthy and sustainable diets: consumer poll. 2021. Accessed April 2026. https://www.food.gov.uk/research/wider-consumer-interests/healthy-and-sustainable-diets-consumer-poll.

[54] McKinsey & Company. Hungry and confused: the winding road to conscious eating. 2022. Accessed April 2026. https://www.mckinsey.com/industries/consumer-packaged-goods/our-insights/hungry-and-confused-the-winding-road-to-conscious-eating#/.

[55] Van Loo EJ , Diem MNH , Pieniak Z , et al. Consumer attitudes, knowledge, and consumption of organic yogurt. J Dairy Sci. 2013;96:2118–2129. 23415537 10.3168/jds.2012-6262

[56] Kresova S , Gutjahr D , Hess S. German consumer evaluations of milk in blind and nonblind tests. J Dairy Sci. 2022;105:2988–3003. 35151472 10.3168/jds.2021-20708

[57] Shortall O. Cows eat grass, don’t they? Contrasting sociotechnical imaginaries of the role of grazing in the UK and Irish dairy sectors. J Rural Stud. 2019;72:45–57.

[58] Gradidge S , Zawisza M , Harvey AJ , et al. A structured literature review of the meat paradox. Soc Psychol Bull. 2021;16:1–26.

[59] Scott E , Kallis G , Zografos C. Why environmentalists eat meat. PLoS One. 2019;14:e0219607. 31295301 10.1371/journal.pone.0219607 PMC6622546

[60] Howley P , Yadav L , Hynes S , et al. Contrasting the attitudes of farmers and the general public regarding the ‘multifunctional’ role of the agricultural sector. Land Use Policy. 2014;38:248–256.

[61] Farming UK. Two-thirds of consumers unsure what Red Tractor assurance is. 2019. Accessed April 2026. https://www.farminguk.com/news/two-thirds-of-consumers-unsure-what-red-tractor-assurance-is_53644.html.

[62] Compassion in World Farming. Farm assurance schemes and animal welfare: how the standards compare. 2012. Accessed April 2026. https://www.ciwf.org.uk/media/5231246/standards_analysis_exec_summary.pdf.

[63] Gorton M , Yeh C–H , Chatzopoulou E , et al. Consumers’ willingness to pay for an animal welfare food label. Ecol Econ. 2023;209:107852.

[64] Kenny TA , Woodside JV , Perry IJ , et al. Consumer attitudes and behaviors toward more sustainable diets: a scoping review. Nutr Rev. 2023;81:1665–1679. 37014671 10.1093/nutrit/nuad033 PMC10639109

[65] Office for National Statistics. Consumer trends, UK. July to September 2025. Accessed April 2026. https://www.ons.gov.uk/economy/nationalaccounts/satelliteaccounts/bulletins/consumertrends/julytoseptember2025/relateddata.

