# Supplementary material for: Balancing nutrition, ethics, and sustainability about dairy: UK consumers’ knowledge, attitudes, practice, and intended choices
Source: J Nutr Sci. 2026 Jul 7;15:e51. doi: 10.1017/jns.2026.10109 (PMC13369254; doi:10.1017/jns.2026.10109)
Supplement: Bracey et al. supplementary material 1 — Bracey et al. supplementary material [file S2048679026101098sup001.pdf]

Dairy and You Consumer Survey

This questionnaire is designed to explore your knowledge and attitudes of the UK dairy industry and dairy products and examine how that impacts purchase decisions. Take your time to read the questions carefully before answering. If you have any queries while filling the questionnaire, please contact a member of the research team.

Section 1 – Knowledge

How much do you know about dairy and dairy farming in the UK? Please answer as many questions as possible, as we are keen to understand how much people know.

Q1.What do cows typically eat in the UK?

Maize ☐
Grass (fresh or preserved) ☐
Wheat ☐
Soybean ☐
Waste or unwanted foods ☐
Don't know ☐

Q2. Whilst it varies farm to farm, how many times daily are cows typically milked?

Once ☐
Twice ☐
Whenever the cow wants ☐
Four ☐
Six ☐
Don't know ☐

Q3. What gas do cows 'burp' that is linked with climate change?

Helium ☐
Methane ☐
Oxygen ☐
Nitrous Oxide ☐
Argon ☐
Don't know ☐

Q4. One key issue in cow health management an inflammation of the udder. Can you name this condition?

- Cowpox ☐
- Foot & Mouth ☐
- Bovine Spongiform Encephalopathy (BSE) ☐
- Mastitis ☐
- Bovine Tuberculosis ☐
- Don't know ☐

Q5. Cows are typically housed in barns for periods of time throughout the year. How many days a year do you think the average UK dairy cow spends outside?

- Less than 75 ☐
- 76 to 150 ☐
- 151 to 225 ☐
- 226 to 300 ☐
- 300+ ☐
- Don't know ☐

Q6. 'Cow comfort' is an important factor for cow health. What is the one main factor that dictates cow comfort?

- Regular massages ☐
- Music playing ☐
- Light in the barn ☐
- Type of feed ☐
- The bedding and lying space ☐
- Don't know ☐

Q7. How many litres of milk a year does the average UK dairy cow produce?

- 5200 ☐
- 3700 ☐
- 12000 ☐
- 10200 ☐
- 8000 ☐
- Don't know ☐

Q8. Much of the milk consumed undergoes a process of heating to ensure milk is safe to consume. What is the name of this process?

Ergotisation ☐
Pasteurization ☐
Homogenisation ☐
Chiralisiation ☐
Purification ☐
Don't know ☐

Q9. Homogenisation is a process some milks undergo, but what is the purpose of this process?

A process that heat treats the milk to ensure a long-life ☐
A process to add nutrients to the milk ☐
A process to prevent spoiling of milk ☐
A process by which the cream does not separate from the milk. ☐
A process that turns the milk white ☐
Don't know ☐

Q10. Cow slurry (cow manure), is also a concern for climate change but why?

Decomposes and releases nitrous oxide during storage ☐
When spread on fields it can make plants and crops grow too fast ☐
Can leech into rivers and kill aquatic life. ☐
Need space to store it ☐
Needs to be transported for disposal ☐
Don't know ☐

Q11. The following nutrients, found in dairy, play a role in health. Can you identify which nutrient influences each bodily function? (Tick one box each row)

	**Calcium**	**Iodine**	**Protein**	**Vitamin A**	**Don't know**
Bone health					
Thyroid function					
Vision					
Maintain muscle mass or grow					

Q12. Of the following nutrients listed, would you consider cow's milk to be a good source of

	Not a source	Is a Source	Don't know
Iron			
Calcium			
B12			
Iodine			
Vitamin D			
Folic Acid			
Omega-3 fats			
Omega-6 fats			

The following are a list of quality assurance brands that regularly appear on many dairy products.

Red tractor
Soil association
RSPCA Assured
Lion Mark
Fairtrade

Q13. Have you seen or heard of them before?

Yes, all of them ☐
Yes, some ☐
No

Q14. Can you identify the following quality assurance brands that regularly appear on many dairy products?

Section 2 – Your opinions about dairy

**Q15. The dairy industry in the UK….**	**Strongly agree**	**Agree**	**Neutral**	**Disagree**	**Strongly disagree**	**Don't know**
Is not a major producer of carbon dioxide and methane emissions						
Is a major source of nitrogen emissions						
Is doing significant damage to the land						
Is good for the environment						
Is not an important factor in ensuring the UK produces its own food.						
Produces safer and higher quality food than in Europe						

How strongly do you agree or disagree with the following statements? (Please tick one box in each row)

**Q16. Dairy processors in the UK…**	**Strongly agree**	**Agree**	**Neutral**	**Disagree**	**Strongly disagree**	**Don't know**
Are dominated by large companies						
Do not pay a fair price to their farm suppliers.						
Want to create nutritious products for their customers						
Support farmers to produce nutritious dairy products						

**Q17. Dairy farmers in the UK…**	**Strongly agree**	**Agree**	**Neutral**	**Disagree**	**Strongly disagree**	**Don't know**
care for their animals/cows.						
do not effectively manage herd health issues.						
ensure that herd reproduction practices prioritise the welfare of both the calf and the cow.						
do not responsibly manage the life outcomes of calves.						
ensure their cows are living healthy and happy lives.						

Q18. Dairy farmers in the UK…

**Q18. Dairy farmers in the UK…**	**Strongly agree**	**Agree**	**Neutral**	**Disagree**	**Strongly disagree**	**Don't know**
ensure their cows have a high-quality diet.						
Ensure their cows have plenty of access to outdoor space						
ensure the milk they produce is safe.						
ensure the milk they produce is nutritious.						

**Q19. Dairy farmers in the UK…**	**Strongly agree**	**Agree**	**Neutral**	**Disagree**	**Strongly disagree**	**Don't know**
contribute to the local and regional food landscape						
help ensure UK food security						
are getting a good price for their milk						
produce a key food to ensure people have a healthy balanced diet						

Strongly Agree	Agree	Somewhat agree	Neutral	Somewhat disagree	Disagree	Strongly disagree

Q20. How strongly do you agree or disagree you are consuming a healthy balanced diet?

Q21. Do you think that plant-based milk is a nutritionally adequate alternative to cow's milk?

Yes ☐
No ☐
Don't know ☐

Q22. List three words you would associate with dairy foods in the UK (e.g. milk, cheese etc).

Q23. List three words you would associate with the UK dairy industry.

Q24. As a consumer are there any other comments you would like to add about dairy farming, cow welfare, production, or environmental impact in the UK?

Section 3 - Improving milk and dairy

Q25. Some people in the UK have insufficient intakes of nutrients such as vitamin D, iodine, selenium and omega-3 fats. It is possible to produce milk with these nutrients in them through adding new nutritionally interesting crops to the diet of the cows. Please state 'how likely you would consider' each of the following statements…

	Extremely unlikely	Unlikely	Neutral	Likely	Extremely likely	N/A
a. trying improved milks if they were to positively impact your health?						
b. trying improved milks if they were to improve the health of others in your household, such as children?						
c. trying improved milks if the feed used also improved the health and welfare of the cows?						
d. trying improved milks if the feed used also reduced the environmental impact of the dairy industry?						
e. purchasing dairy products that improved human and animal health, and reduced environmental impact?						

Q26. It is also possible to fortify the milk at the point of processing, without any benefits to factors such as reduced environmental impact or cow health. Which method would you prefer?

Through cow diet ☐
Through processing ☐
Neither ☐

Section 4 – Your dairy consumption

Please tell us about your dairy consumption habits.

Q27. How frequently do you drink or consume any of the following types of animal or plant-based milk. (Tick all that apply)

	Multiple times daily	Daily	Weekly	Monthly	Occasionally (less than once a month)	Do not drink or consume	Don't know
Cow							
Goat							
Sheep							
Soy							
Almond							
Rice							
Oat							

If answer to Q27 is 'Do not drink or consume' cows milk selected, skip to section 5.

Q28. What type of cow's milk do you mainly drink or use? (Tick one)

	You	other people in your household
Full fat	☐	☐
Semi-skimmed	☐	☐
Skimmed	☐	☐
Do not drink or purchase cow's milk	☐	☐

Q29. If you drink COW's milk, how often and how do you drink it?

Tick one column for each food (per day, per week or per month)

	Per day (servings)				Per week			Per month	
	6+	4-5	2-3	1	5-6	2-4	1	1-3	<1
With tea/ coffee (other than cappucine/latte)									
On breakfast cereals									
Milk for chocolate/ horlicks drink/ cappuccino/latte									
Just as it is (i.e glas/ cup of milk)									

Q30. In your typical diet, how often do you eat these foods?

Tick one column for each food (per day, per week or per month)

	Per day (servings)				Per week			Per month	
	6+	4-5	2-3	1	5-6	2-4	1	1-3	<1
Cheese (hard or soft)									
Cheese based dishes (i.e. pizza, sandwich, cauliflower cheese)									
Yoghurts									
Milk or cream-based puddings (e.g. custard, ice cream)									

Q31. How often does your household purchase cow's milk from the following outlets?	Never	Rarely	Sometimes	Often	Always
Supermarket (Tesco/Asda/Morrisons/Sainsburys/ etc.)					
Specialist food store (Delicatessen etc.)					
Local independent shop					
Market (Farmers market etc.)					
Farm shop					
Milkman/milk delivery					

Q32. How often do you or your household purchase the following cows milk products?	Never	Rarely	Sometimes	Often	Always
Retailer own brand (Tesco/Asda/Lidl/etc)					
Branded					
Organic					
Jersey					
A2					
Microfilter/Purefilter					
UHT or Long-life					
Unhomogenised					
Raw / unpasteurised					
Best of Both skimmed milk with added protein)					
With added health benefits (Eg. with added plant sterols)					
Lactofree					

Always	Very often	Sometimes	Rarely	Never

Q33. How often do you look for quality assurance brands, such as Red Tractor, when purchasing dairy products?

**Q34. What other types of dairy products made from cow's milk does your household regularly purchase?**	**Never**	**Rarely**	**Sometimes**	**Often**	**Always**
Hard Cheese (cheddar/blue cheese or equivalent)					
Soft Cheese (cream cheese/Brie or equivalent)					
Yoghurt					
Cream (Single/double/whipping etc)					
Crème fraiche/sour cream/clotted/custard/buttermilk/					
Butter or other spreads containing buttermilk					
Ice cream					

**Q35. How often does your household purchase cow's milk dairy products (such as cheese) from the following outlets?**	**Never**	**Rarely**	**Sometimes**	**Often**	**Always**
Supermarket (Tesco/Asda/Morrisons/Sainsburys/ etc.)					
Specialist food store (Delicatessen etc.)					
Local independent shop					
Market (Farmers market etc.)					
Farm shop					
Milkman/milk delivery					

Q36. Excluding high and low fat dairy products, do you regularly and intentionally purchase other dairy products with health or nutrition claims to improve your nutritional health (e.g. low cholesterol or high in beta-carotenoids)?

Always	Very often	Sometimes	Rarely	Never

Q37. When purchasing milk how likely do you think you look at the following items on the packaging before deciding on your purchase?

	Very likely	Likely	Neutral	Unlikely	Very unlikely	Don't know
Label						
Nutritional information						
Price						
Quality assurance (e.g. Red Tractor, Soil Association)						
Nutrition claims						
Health claims						

Q38. Consider the following scenario - Four new ranges of milk have appeared on the shelves of your regular retail outlet. You see that there are distinct differences between each brand. How of likely would you purchase the following options? (See table below)

	Definitely	Very Probably	Probably	Possibly	Probably Not	Definitely Not	Don't know
**Product A**							
**Product B**							
**Product C**							
**Product D**							

	Product A	Product B	Product C	Product D
**Fortification**	States it is fortified with iodine, vitamin D, selenium, & omega 3 during processing.	States the cows have been fed a diet high iodine, vitamin D, selenium, & omega-3 naturally enriching the milk.	No special fortification or enrichment.	States the cows have been fed a diet high iodine, vitamin D, selenium, & omega-3 naturally enriching the milk.
**Health & Welfare claims**	No animal health or welfare claims.	Claims to improve animal health and welfare.	No animal health or welfare claims.	Claims to improve animal health and welfare.
**Environment**	No environmental claims.	Claims reduced environmental impact.	No environmental claims.	Claims reduced environmental impact.
**Location of production**	States it is produced locally or regionally.	No source of production labelling.	No source of production labelling.	States the farm produced and location processed.
**Quality Assurance**	Standard quality assurance label.	Standard quality assurance label.	Standard quality assurance label.	New quality assurance brand.
**Fair trade**	No statement of price paid to farmers.	No statement of price paid to farmers.	No statement of price paid to farmers.	Clear statement it pays a fair price to the farmer.
**Cost**	Costs 80p/litre	Costs 90p/litre	Costs 70p/litre	£1/litre

Q39. In the previous question we asked you to select what type of milk you would purchase. Thinking about your answer, what were the factors that influenced your decision? (Please rank from 1 to 7 each option. With 7 being most important and 1 the least important)

	1	2	3	4	5	6	7
Improved nutritional value of the milk							
Improved animal health or welfare							
Reduced environmental impact							
Source of production							
Quality assurance label							
Farmers’ fair pay							
Price							

Section 5 – About You

Q40. What is your gender?

Male ☐
Female ☐
Other ☐
Prefer not to say ☐

Q41. What is your age? ☐

Q42. What is your ethnic background?

White British (Scottish, English, Welsh, Northern Irish) ☐
African British ☐
Indian British ☐
Other Asian British ☐
Other Europeans ☐
Asian ☐
African ☐
Latin American ☐
American ☐
Hispanic ☐
Mixed ethnicity ☐
Other ☐
If you selected other, please specify ☐

Q43. What is your post code area? (Please state the first half of your postcode e.g G1, EH20, DE7) ☐

Q44. What is the highest level of education you have completed?

Secondary Education (GCSE or equivalent) ☐
Further Education (College/Sixth form) (A levels, Highers, HNC, SVQ, or equivalent) ☐
Higher education – Undergraduate degree (Bachelors) ☐
Higher education postgraduate degree (Masters/ PhD) ☐
Did not complete secondary education ☐
Prefer not to say ☐

Q45. Have you studied any of the following subjects post secondary education?

Nutrition / Dietetics ☐
Food Sciences / Food Technology / Food Chemistry (or similar) ☐
Medicine / Dentistry / Nursing ☐
Health Sciences (pharmacy, midwifery, other allied health professions) ☐
Farming / Agriculture ☐
Other ☐
Prefer not to say ☐

Q46. How many people 18 years or over live in your household? ☐

Q47. How many people 17 years or younger live in your household? ☐

Q48. What is your current employment status?

Full-time employment (30+ hours/week) ☐
Part-time employment (less than 30 hours/week) ☐
Unemployed (any reason) ☐
In further education or training ☐
Prefer not to say ☐

Q49. What is your total combined household income from all sources before tax, national insurance, pensions and so on?

Up to £20,000 ☐
£20,001 to £40,000 ☐
£40,001 to £60,000 ☐
£60,001 to £80,000 ☐
£80,001 and above ☐
Prefer not to say ☐

Q50. Do you currently follow a particular diet?

Yes ☐
No ☐

Q51. If Yes (to Q51),

For weight loss ☐
For other reasons (e.g. ethics, medical, personal choice) ☐

Q52. If Yes (to Q52), which particular diet?

Lacto-Vegetarian (Just dairy) ☐
Ovo-vegetarian (Just eggs) ☐
Lacto-ovo-vegetarian (eggs & dairy) ☐
Vegan ☐
Pescatarian ☐
Flexitarian ☐
Low Carb diet ☐
Paleo ☐
Dukan ☐
Ultra-low fat ☐
Atkins ☐
Intermittent fasting ☐
Ketogenic ☐
Weight Watchers ☐
Raw food ☐
Mediterranean ☐
Religious ☐
Prefer not to say ☐
Other

Q53. Have you been told by your GP / doctor that you have any of the following?

Diabetes (type 1) or (type 2) ☐
Heart Disease ☐
Obesity ☐
Inflammatory bowel syndrome ☐
Anaemia from iron deficiency ☐
High blood pressure ☐
High blood cholesterol/triglyceride ☐
Irritable bowel syndrome ☐
Osteoporosis ☐
Sarcopenia ☐
Other ☐

Q54. Are you allergic to lactose?

Yes ☐
No ☐

If yes, was this diagnosed by a GP / doctor? ☐ Yes ☐ No

Q55. How physically active are you? NB: 'raising heart rate' means unable to withhold a conversation at that intensity of physical activity.

Fairly inactive (walking only) ☐
Moderately active (occasionally take exercise, that raise my heart rate, less than 3 times per week) ☐
Very active (regularly take exercise, that raise my heart rate, 3 times a week or more) ☐

Q56. Have you had any falls in the past 5 years? e.g. a fall to the ground/fall down the stairs.

Yes ☐
No ☐

Q41. If yes, how many? ☐

Q57. Have you had any bone fractures in the past 5 years?

Yes ☐
No ☐

Q58. If yes, how many? ☐

Q59. Are you the main grocery shopper in your household?

Yes ☐
No ☐

Q60. Are you the main cook in your household?

Yes ☐
No ☐

Q61. How much does your household spend on food each week? (Best approximate guess)

£0-50 ☐
£50-99 ☐
£100-149 ☐
£150 – 199 ☐
£200-249 ☐
£250-299 ☐
Over £300 ☐
Don't know / prefer not to say ☐

Before you close the survey

Please provide us contact details in the box below (contact email address) if you would like to enter the prize draw. This will be detached from your filled questionnaire to anonymise your data.

If you would also like to find out about our research findings from this survey and for future opportunities to participate in our research, please tick the following box. ☐

Email -
