# Supplementary material for: Balancing nutrition, ethics, and sustainability about dairy: UK consumers’ knowledge, attitudes, practice, and intended choices
Source: J Nutr Sci. 2026 Jul 7;15:e51. doi: 10.1017/jns.2026.10109 (PMC13369254; doi:10.1017/jns.2026.10109)
Supplement: Bracey et al. supplementary material 2 — Bracey et al. supplementary material [file S2048679026101098sup002.docx]

Supplementary information

## Table 1. Descriptive statistics comparing high and low consumer groups, raw and Benjamin-Hochberg adjusted p-values

| **Variable** | **Raw p-value** | **Adjusted p-value** |
| --- | --- | --- |
| Sex | 0.1398 | 0.183488 |
| Age | 0.6368 | 0.6368 |
| Ethnicity (white British) | 0.06477 | 0.104628 |
| Urban / Rural | **1.95E-05** | **0.000355** |
| Household IMD | **0.03966** | 0.069405 |
| Household income | 0.5037 | 0.528885 |
| Education | **0.02206** | **0.042115** |
| *Below degree* | **0.02071** | **0.042115** |
| *Postgraduate* | **0.009974** | **0.023273** |
| Subject studied | **0.000837** | **0.00293** |
| *farming* | **0.001156** | **0.003468** |
| Employment | 0.5557 | 0.5557 |
| Dietary Pattern | **5.07E-05** | **0.000355** |
| *Omnivore* | **0.008932** | **0.023273** |
| *Vegetarian* | **3.97E-05** | **0.000355** |
| Main shopper | 0.07248 | 0.10872 |
| Main cook | 0.128 | 0.1792 |
| Health | 0.1512 | 0.186776 |
| Activity level | **0.000425** | **0.001786** |
| *Very active* | **0.000328** | **0.001723** |
| History of falls | 0.1953 | 0.22785 |
| History of fractures | 0.2262 | 0.250011 |

## Table 2. Summary of ANCOVA models for cumulative attitude scores, comparing differences between high and low consumption groups, adjustments made based on variables making significant contributions to the initial model.

|  | *F* | Degrees of Freedom | P-value | R^2^ | Adjusted R^2^ |
| --- | --- | --- | --- | --- | --- |
| Animal welfare^1^ | 17.67 | 11, 660 | <0.001 | 0.238 | 0.222 |
| Food security^2^ | 15.84 | 11, 669 | <0.001 | 0.207 | 0.194 |
| Nutrition^3^ | 25.11 | 7, 670 | <0.001 | 0.208 | 0.200 |
| Sustainability^4^ | 21.84 | 11, 661 | <0.001 | 0.267 | 0.254 |
| ^1^Adjustments made for gender, dietary pattern, age, urban/rural, education and subject studied  ^2^Adjustments made for dietary pattern, age, urban/rural, education and subject studies  ^3^Adjustments made for dietary pattern, age, urban/rural and education  ^4^Adjustments made for dietary pattern, age, urban/rural, education and subject studied | | | | | |

## Table 3. Means and adjusted means of each model for cumulative attitude scores comparing differences between high and low consumption groups.

|  | Unadj. mean (SD) | | Unadj. group diff. | Unadj. p-value | Adj. mean (SD) | | Adj. group diff. | Adj. p-value |
| --- | --- | --- | --- | --- | --- | --- | --- | --- |
|  | Low | High | Δ |  | Low | High | Δ |  |
| *Domain 1: animal welfare^1^* | 77.1 (0.83) | 88.6 (1.01) | 11.5 | <0.0001 | 76.0 (1.85) | 82.6 (1.91) | 6.6 | 0.1704 |
| *Domain 2: food security^2^* | 84.9 (0.65) | 92.9 (0.80) | 8.02 | <0.0001 | 83.5 (1.56) | 89.7 (2.19) | 6.2 | 0.0152 |
| *Domain 3: nutrition^3^* | 76.0 (0.60) | 84.0 (0.74) | 7.99 | <0.0001 | 71.6 (1.20) | 78.6 (1.32) | 6.95 | <0.0001 |
| *Domain 4: environment^4^* | 63.5 (0.99) | 78.0 (1.22) | 14.5 | <0.0001 | 67.7 (2.14) | 72.1 (2.48) | 4.38 | 0.0007 |
| ^1^Adjustments made for gender, dietary pattern, age, urban/rural, education and subject studied  ^2^Adjustments made for dietary pattern, age, urban/rural, education and subject studies  ^3^Adjustments made for dietary pattern, age, urban/rural and education  ^4^Adjustments made for dietary pattern, age, urban/rural, education and subject studied | | | | | | | | |

## Table 4. Summary of initial multivariable logistic regression model for preference for Product D (binary)

| Variable | Odds Ratio (OR) | 95% Confidence Intervals | | P-value |
| --- | --- | --- | --- | --- |
|  |  | Lower | Upper |  |
| *Intercept* | 4.01 | 0.28 | 59.4 | 0.308 |
| High/low consumption^1^ | 0.41 | 0.22 | 0.76 | **0.005** |
| Household income^2^ | 1.01 | 0.82 | 1.23 | 0.950 |
| Age^3^ | 1.00 | 0.98 | 1.02 | 0.976 |
| Gender^1^ | 0.65 | 0.37 | 1.14 | 0.136 |
| Children in household^3^ | 0.97 | 0.75 | 1.24 | 0.826 |
| IMD^2^ | 0.94 | 0.81 | 1.09 | 0.421 |
| Urban/Rural^1^ | 0.67 | 0.37 | 1.20 | 0.178 |
| Importance of price in habitual purchasing^2^ | 0.78 | 0.61 | 0.98 | **0.038** |
| Cumulative attitude scores: | | | | |
| *- Animal welfare^3^* | 1.03 | 1.01 | 1.05 | **0.010** |
| *- Environment^3^* | 0.98 | 0.96 | 1.00 | **0.016** |
| *- Nutrition^3^* | 0.98 | 0.95 | 1.01 | 0.250 |
| Willingness to try new products^*^: | | | | |
| *- Animal welfare^2^* | 0.67 | 0.42 | 1.07 | 0.101 |
| *- Environment^2^* | 1.20 | 0.76 | 1.92 | 0.450 |
| - *Personal health^2^* | 0.98 | 0.67 | 1.47 | 0.931 |
| - *Family’s health^2^* | 0.86 | 0.59 | 1.22 | 0.403 |
| Willingness to buy improved product^†^: | | | | |
| *- Animal welfare^2^* | 1.06 | 0.86 | 1.30 | 0.567 |
| *- Environment^2^* | 1.01 | 0.83 | 1.24 | 0.917 |
| *- Nutrition^2^* | 0.92 | 0.80 | 1.05 | 0.231 |
| *- Price^2^* | 1.09 | 0.97 | 1.23 | 0.152 |
| ^1^Categorical variables; ^2^Ordinal variables; ^3^Continuous variables  ^*^Based on response to 5-point Likert questions (Q25)  ^†^Based on response to rank (1-7) of factors underpinning perceived subjective preference (Q39) | | | | |

## Table 5. Summary of final multivariable logistic regression model for preference for product D

| Variable | Odds Ratio (OR) | 95% Confidence Interval (CI) | | P-value |
| --- | --- | --- | --- | --- |
|  |  | Lower | Upper |  |
| *Intercept* | 0.62 | 0.19 | 1.95 | 0.460 |
| Consumption | 0.39 | 0.22 | 0.67 | **<0.001** |
| Importance of price in habitual purchasing | 0.74 | 0.59 | 0.91 | **0.005** |
| Cumulative attitudes scores: | | | | |
| - Animal welfare | 1.03 | 1.01 | 1.05 | **0.008** |
| - Environment | 0.97 | 0.96 | 0.99 | **0.001** |
